# Supplementary material for: Recent Advances in Counterfeit Art, Document, Photo, Hologram, and Currency Detection Using Hyperspectral Imaging
Source: Sensors (Basel). 2022 Sep 26;22(19):7308. doi: 10.3390/s22197308 (PMC9571956; doi:10.3390/s22197308)
Supplement: Supplementary file 1 [file sensors-22-07308-s001.zip › sensors-1904766-supplementary.pdf]

Supplementary Materials

1. Supplementary Materials 1

Supplementary Materials 1 provides further details with regard to the main article including literature search, inclusion criteria, exclusion criteria, study inclusion as well as tables and charts which were not presented in the main article such as accuracy chart, forest plot and Deeks' funnel plot.

1.1 Literature Search

Google scholar was used in searching of researches included in this review. Researches published within last 5 years were considered for this review. Introductory paragraphs of each candidate were manually reviewed to evaluate its relevance to this review, and once deemed relevant, numerical values were obtained when available.

1.2 Inclusion Criteria

- Following criteria was considered in inclusion of researches:
- Written in English
- Published within last 5 years
- Concerns hyperspectral imaging application in artwork authentication, document forgery detection, counterfeit currency detection, hologram authentication and photo authentication.
- Includes definite numerical accuracy
- Published on a journal with the impact factor above 3 and the H-index above 50.

1.3 Exclusion Criteria

- Following criteria was considered in exclusion of the researches:
- Studies without numerical accuracy
- Systematic review, meta-analysis, comments, proceedings, study protocols

1.4 Study Inclusion

Combining results for all 5 classifications, total of 1,402 researches were found upon a search on Google scholar. Upon reviewing their titles and abstracts, only a total of 96 researches were deemed relevant. 60 researches were excluded as it did not meet the inclusion criteria and additional 30 researches were excluded for meeting the exclusion criteria, leaving only 6 researches.7 additional researches were selected from the citations of remaining 6 researches. 11 researches without numerical accuracies were included strictly for the purpose of review.

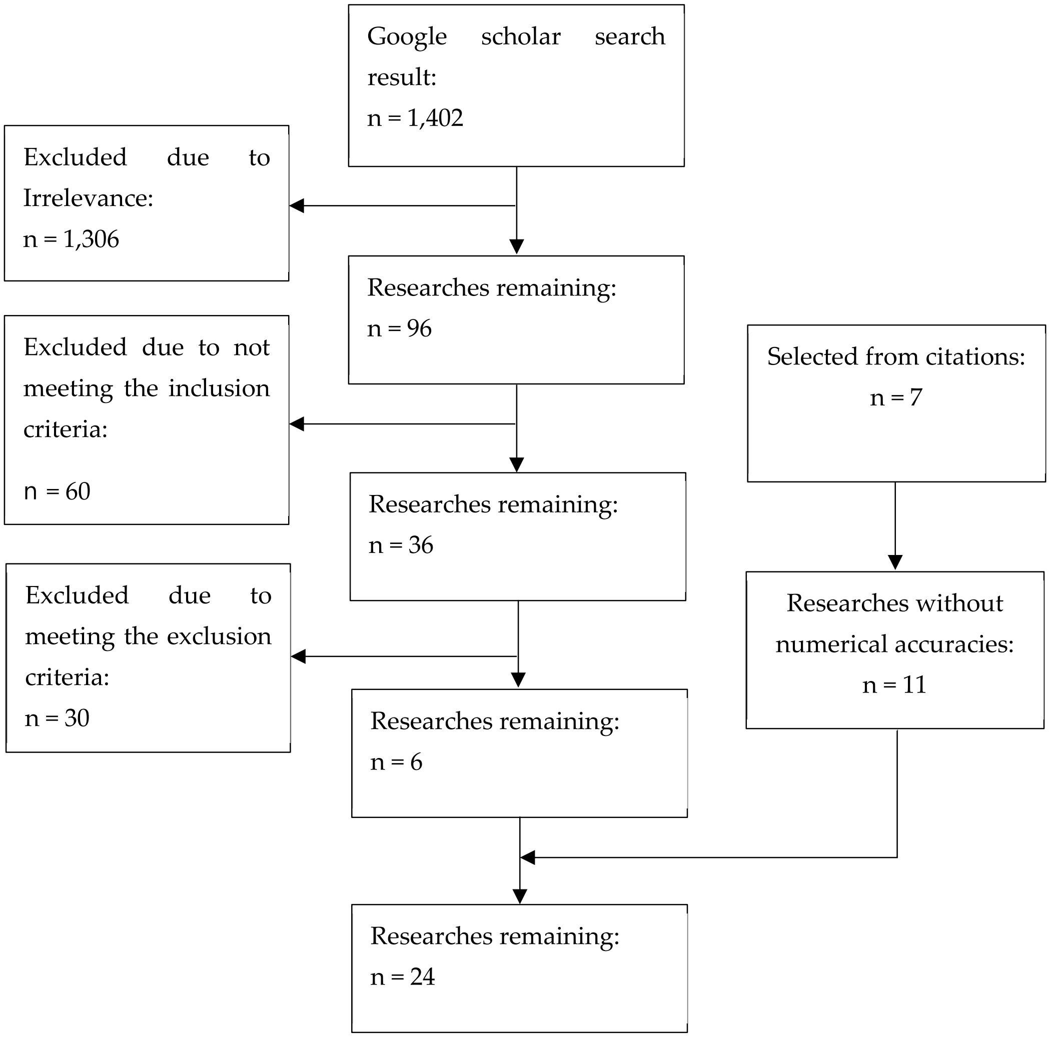

Figure S1. Study Inclusion Flowchart

1.5 Accuracy Charts

Accuracy charts allow visual comparison of given data. This section includes accuracy charts drafted from data acquired from researches included in this review.

Figure S2 shows the accuracy chart of articles classified under artwork. The research done by Grabowski et al. is seen leading the chart with 80.78% accuracy, followed by Wang et al. with 74.03% accuracy and Polak et al. with 72.5% accuracy. Overall accuracy of all researches under this topic classification is 76.42%

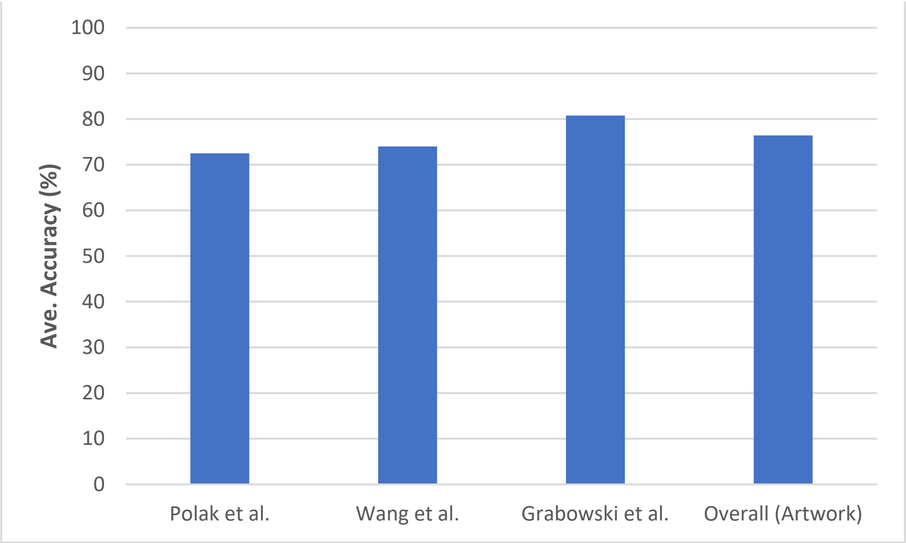

Figure S2. Accuracy Chart of Articles (Artwork)

Figure S3 shows the accuracy chart of articles classified under document. The research done by Khan et al. published in 2018 is seen leading the chart with 93.1% accuracy, followed by Pereira et al. with 87% accuracy, Luo et al. with 85.65% accuracy, another research done by Khan et al. with 81.45%, Silva et al. with 69.67% and finally A. R. Martins et al. with 64% Overall accuracy of all researches under this topic classification is 81.78%.

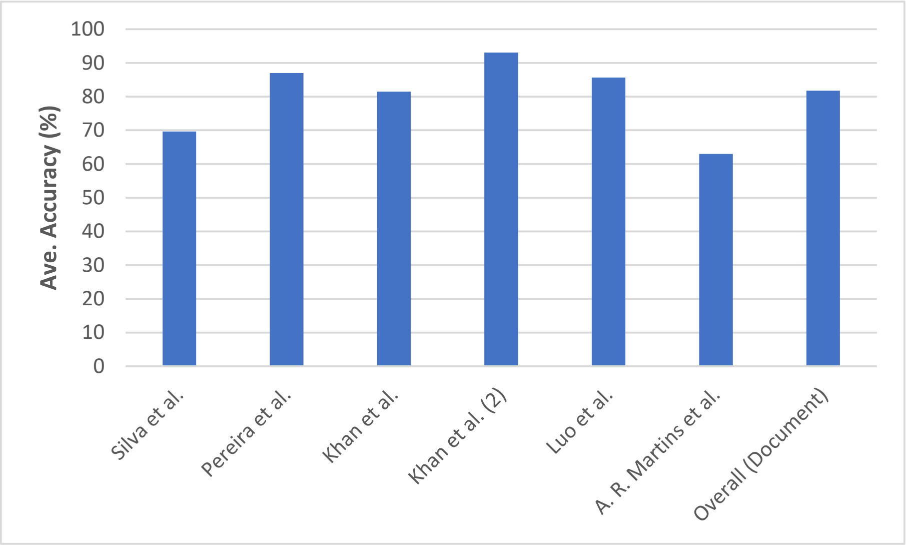

Figure S3. Accuracy Chart of Articles (Document)

Figure S4 shows the accuracy chart of articles classified under currency. The research done by Correia et al. is seen leading the chart with 100% accuracy, followed by Kang et al. with 99.97% accuracy and Baek et al. with 99.28% accuracy. Overall accuracy of all researches under this topic classification is 99.63%

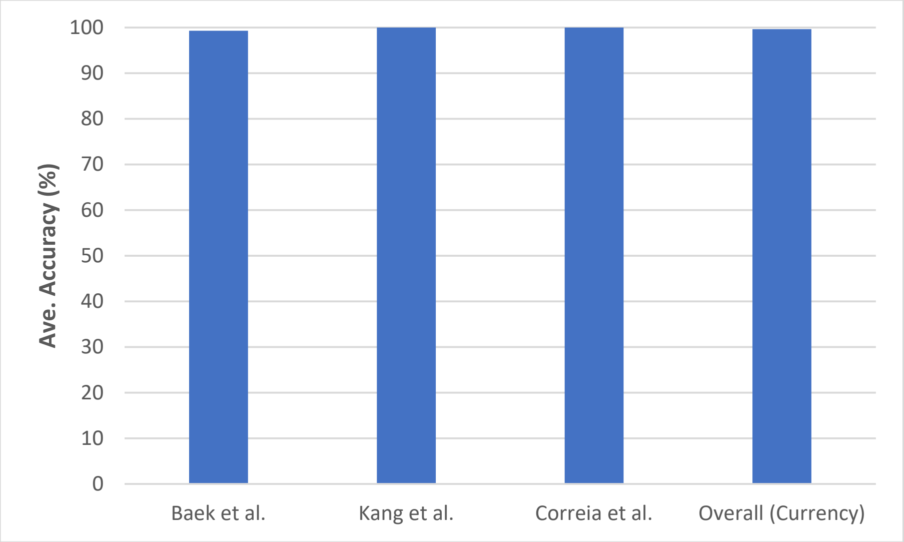

Figure S4. Accuracy Chart of Articles (Currency)

Figure S5 shows the accuracy chart of articles classified under photo. Only one research conducted by Tournié et al. was classified under this topic with 88.27% accuracy. Naturally, overall accuracy of all researches under this topic classification is, therefore, 88.27% as well.

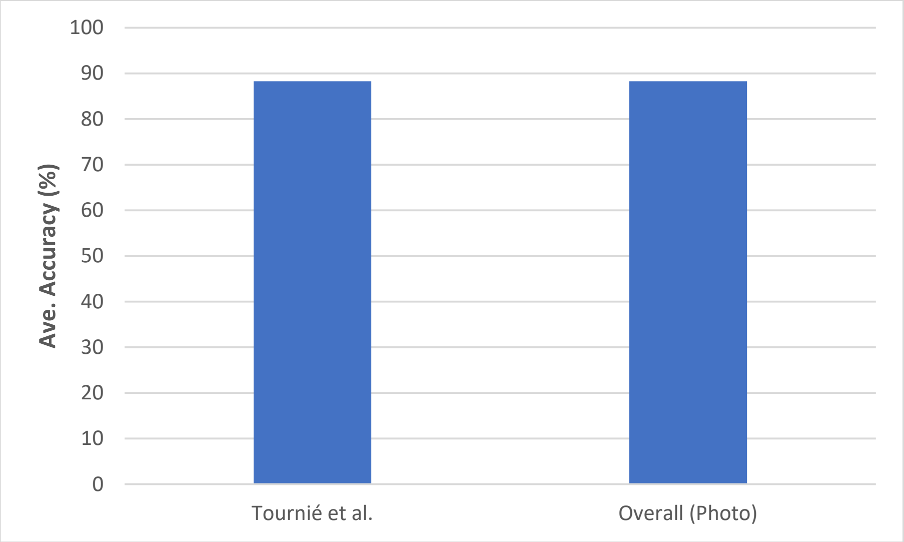

Figure S5. Accuracy Chart of Articles (Photo)

Figure S6 shows the accuracy chart of topic classifications. Among 4 topic classifications, counterfeit currency detection had the highest average accuracy of 99.97%. Followed by photo with 88.27% accuracy and document with 81.78% accuracy. Artwork had the lowest accuracy value of just 76.42%.

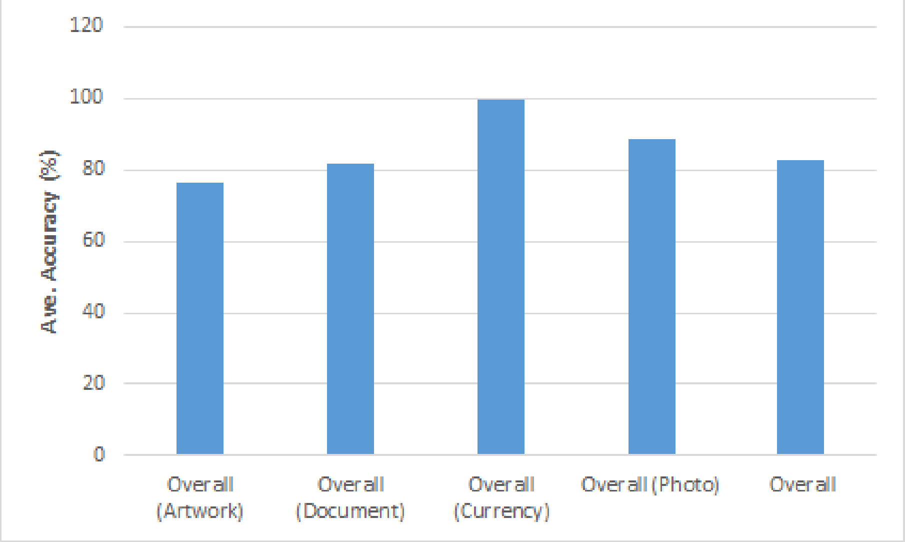

Figure S6. Accuracy Chart of Topic Classifications

Figure S7 shows the accuracy chart of wavelengths. Selecting multiple wavelength ranges yields the highest accuracy of 92.87%, followed by the VIS range with 84.6%, Selecting MIR and SWIR range also yielded decent accuracies of 83% and 83.99% respectively. However, selecting the NIR and VNIR range only yielded accuracies of 78.14% and 76.50%, respectively.

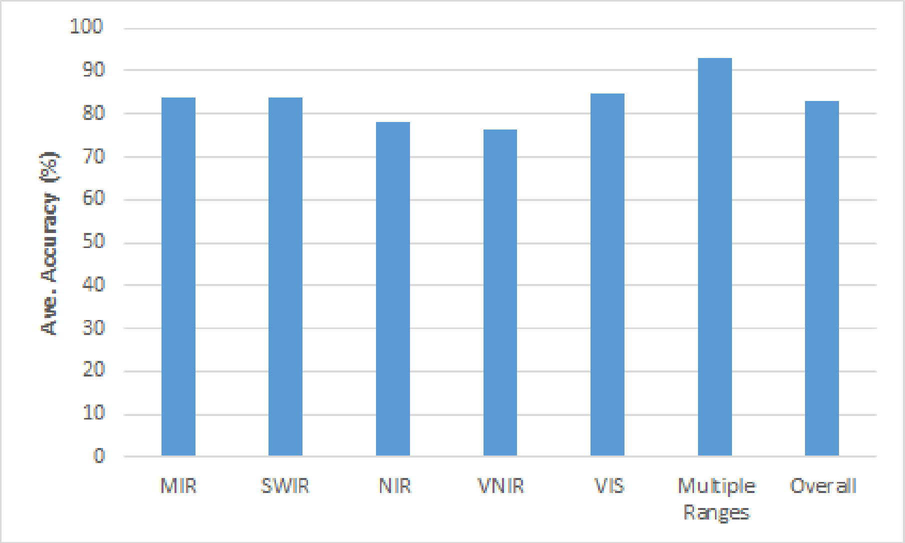

Figure S7. Accuracy Chart of Wavelengths

Figure S8 shows the accuracy chart of processing methods. Based on the data presented herein, dimension reduction process that yields highest accuracy is PP with 90.4% accuracy, followed by SSA with 80.6% accuracy, and PCA with 80.36% accuracy. Meanwhile, the machine learning process that yields the highest accuracy is CNN with 81.53% followed by SVM with 72.5%. Notably, combining multiple processes yielded a higher accuracy of 84.44%.

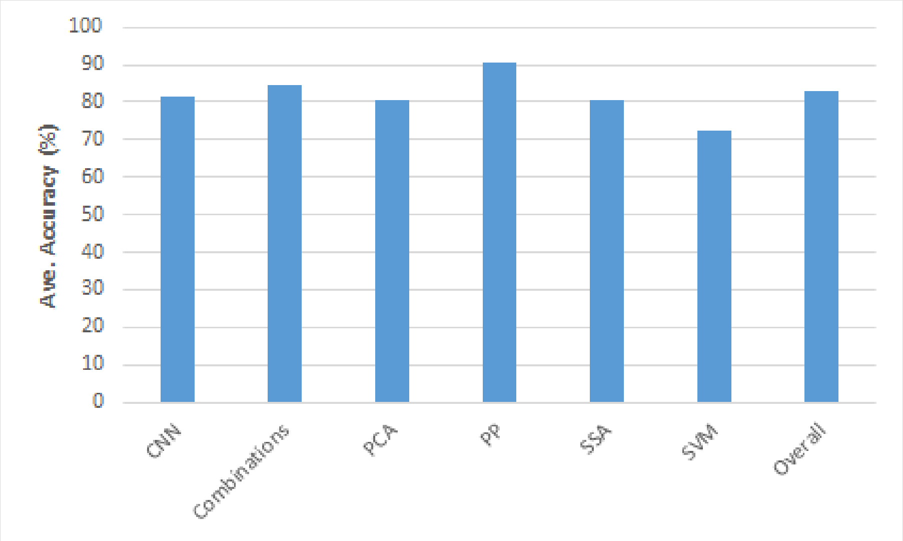

Figure S8. Accuracy Chart of Processing Methods

Figure S9 shows the accuracy chart of year published. Researched published in 2018 has the highest average accuracy of 96.95%, followed by 2016 with 84.30% accuracy, 2015 with 82.85% accuracy, 2017 with 78.02% accuracy, 2014 with 69.67% accuracy, and 2019 with 63% accuracy.

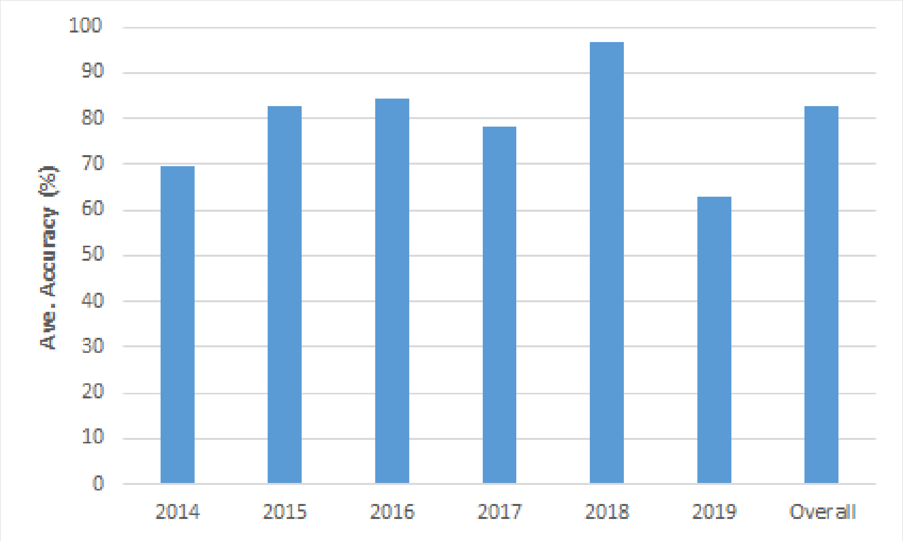

Figure S9. Accuracy Chart of Year Published

1.6 Forest Plots

A forest plot is consisted of data, confidence intervals and the line of no effect. Data is plotted horizontally according to its magnitude and the error bars representing confidence intervals are plotted using these data points as their references. Lastly a vertical line passing through the overall average value is plotted to represent the line of no effect. Any data that overlaps itself or its error bars with this line of no effect is considered as low-performance data. This section includes forest plot drafted from data acquired from researches included in this review.

Figure S10 shows the forest plot of articles classified under artwork. While the research done by Grabowski et al. has the highest accuracy, none of the data, including that of Grabowski et al., is seen displaying high performance.

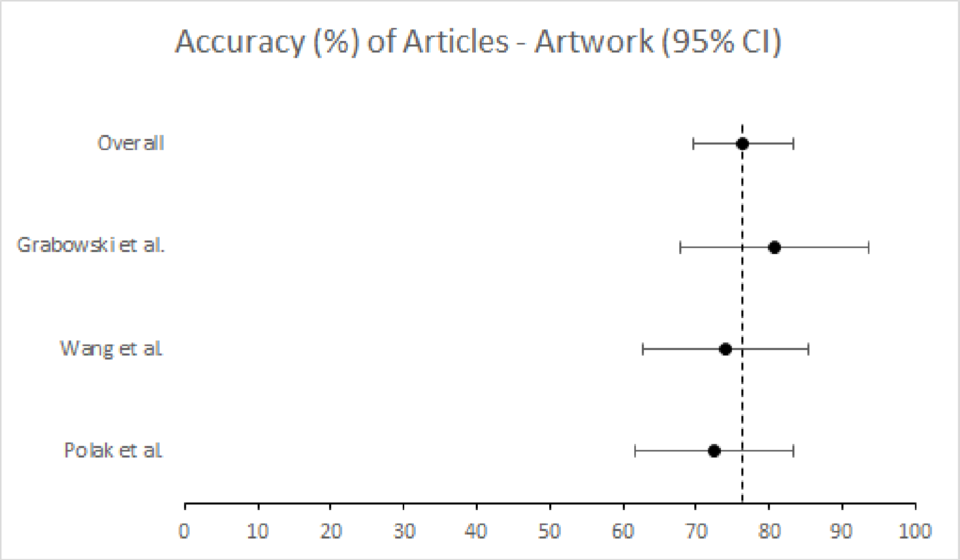

Figure S10. Forest Plot of Articles (Artwork)

Figure S11 shows the forest plot of articles classified under artwork. Only the researches done by Khan et al. in 2018 and A. R. Martins are seen displaying statistical significance.

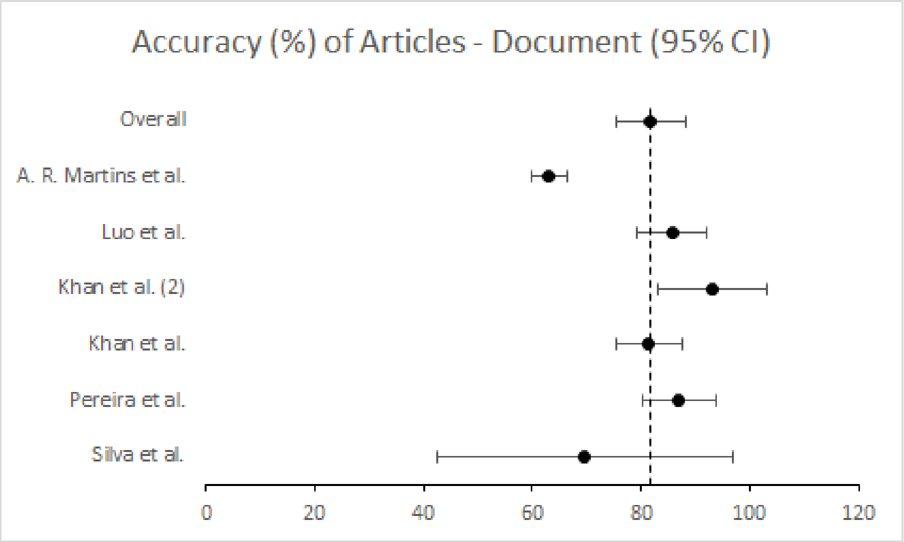

Figure S11. Forest Plot of Articles (Document)

Figure S12 shows the forest plot of articles classified under currency. None of the researches under this topic is seen carrying any statistical significance as they all overlaps with line of no effect.

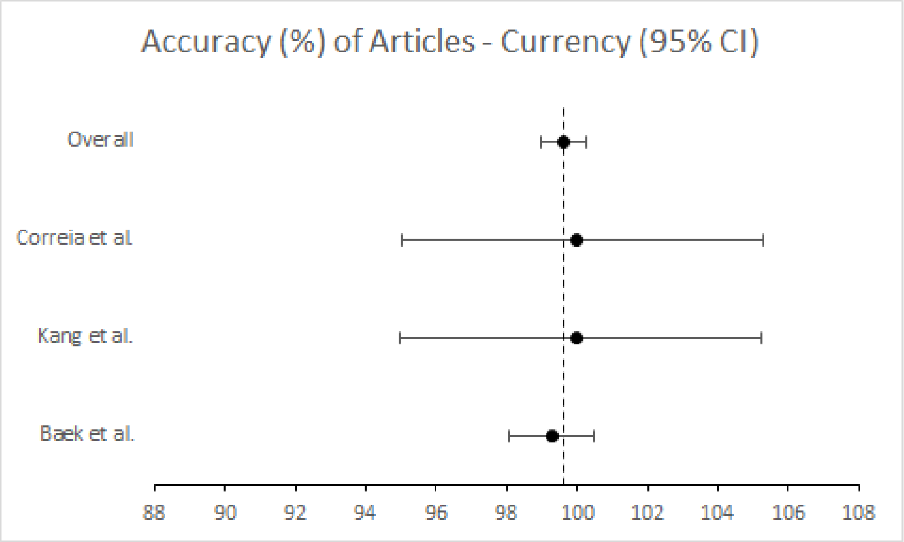

Figure S12. Forest Plot of Articles (Currency)

Figure S13 shows the forest plot of articles classified under photo. Only one research is classified under this topic and therefore it carried no statistical significance.

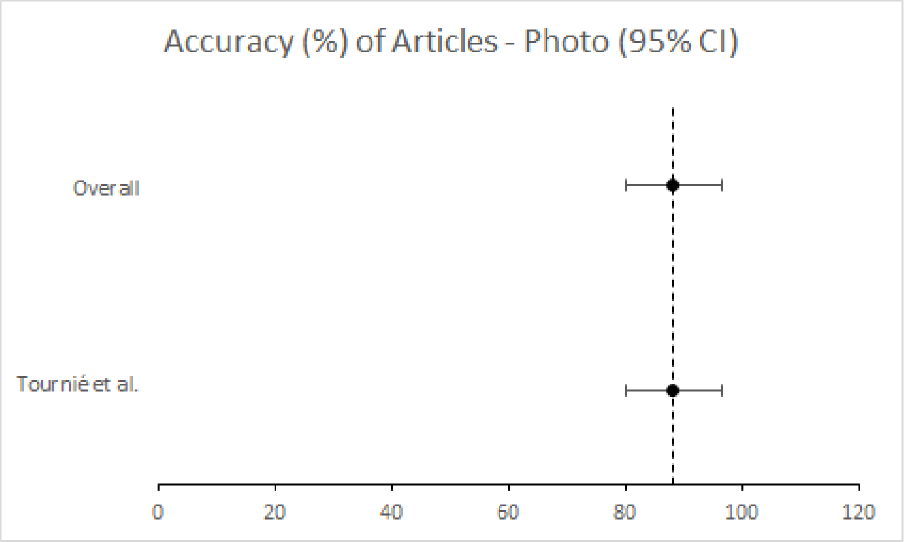

Figure S13. Forest Plot of Articles (Photo)

For topic classification, only studies classified under the topic of counterfeit currency detection were seen carrying statistical significance and displayed outstanding performance with an overwhelming 99.63% average accuracy.

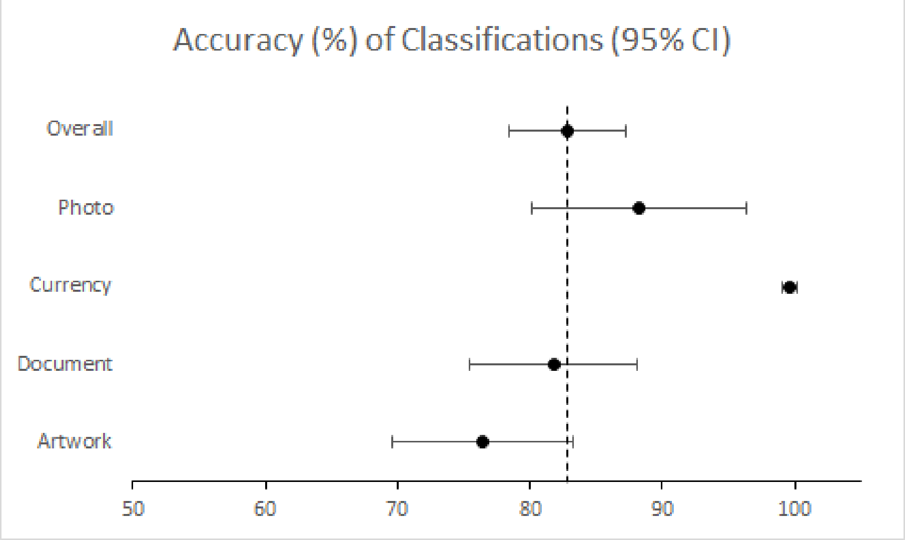

Figure S14. Forest Plot of Topic Classifications

For the wavelengths, only studies that selected multiple ranges showed any statistical significance. It also yielded the best performance with 92.87% average accuracy.

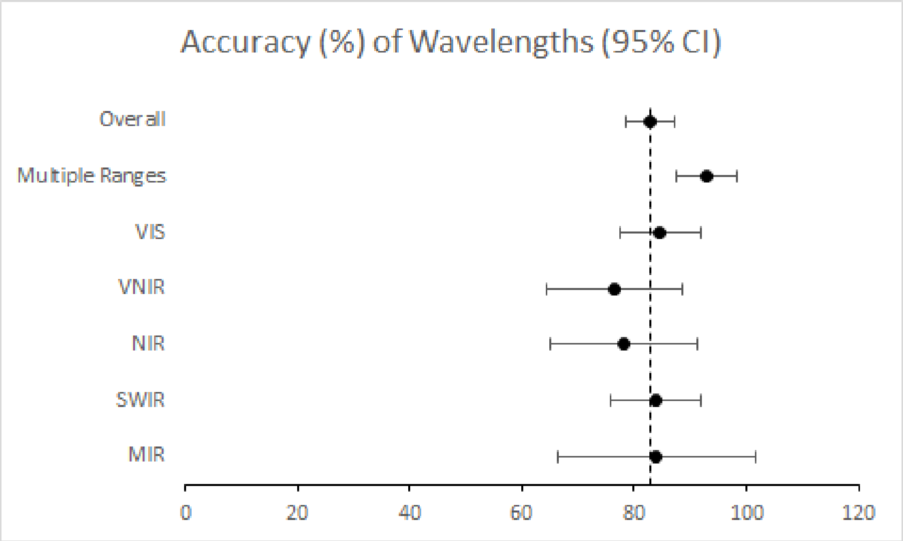

Figure S15. Forest Plot of Wavelengths

Classifying under processing method, no specific method was identified to carry statistical significance because all items were seen overlapping the line of no effect.

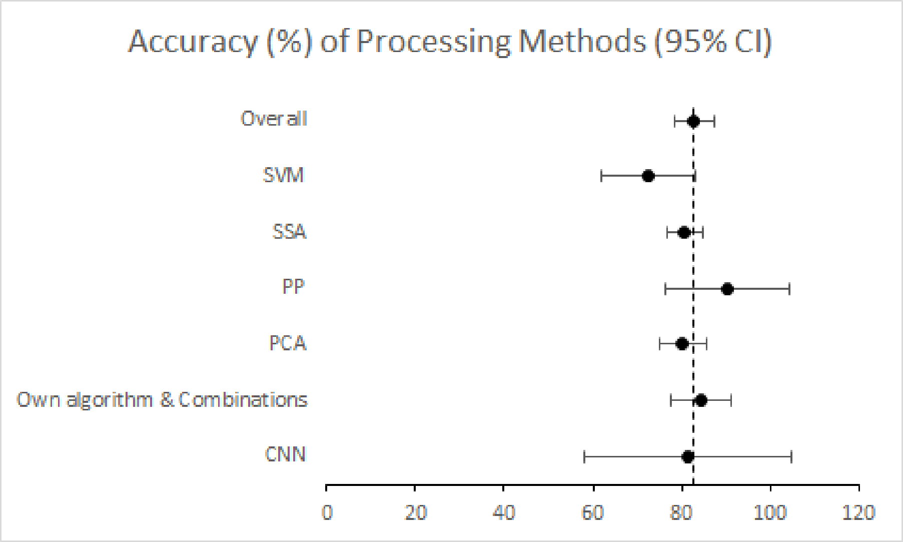

Figure S16. Forest Plot of Processing Methods

When classified under year published, both the studies published in 2018 and those in 2019 were found to have statistical significance. Between both, only the latter performed well with its average accuracy of 96.95%.

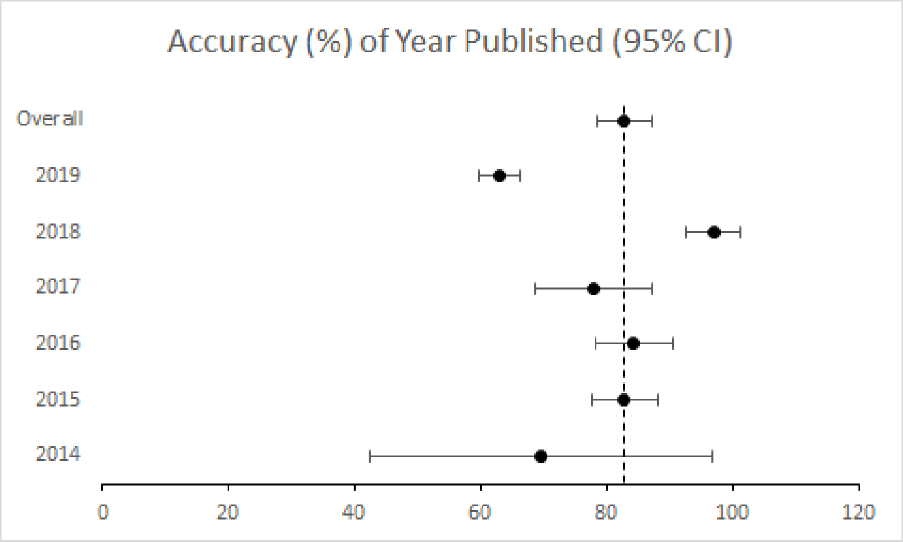

Figure S17. Forest Plot of Year Published

1.7 Deeks' Funnel Plots

Deeks' Funnel Plot is used for the purpose of evaluating publication bias in the field of clinical epidemiology. In this research, a modified version of Deeks' funnel plot that uses accuracies as x-axis values instead of Diagnostic Odds Ratio is employed. This section includes Deeks' funnel plot drafted from data acquired from researches included in this review.

Figure S18 shows the Deeks' plot of articles classified under artwork. The research done by Polak et al., has the smallest sample size as well as the lowest accuracy. The research done by Wang et al, while having the largest sample size, has an accuracy value below the regression line. The research conducted by Grabowski et al. has relatively large sample size while also having high accuracy value.

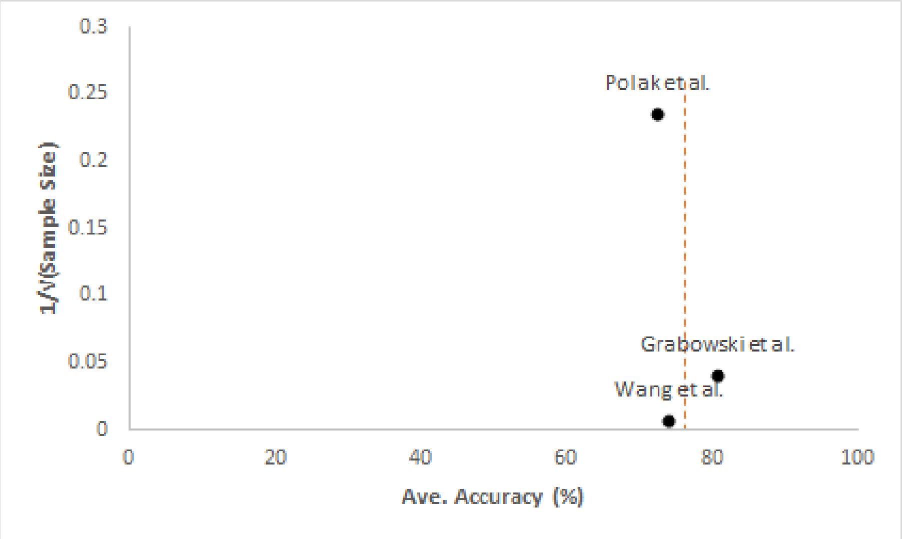

Figure S18. Deeks' Funnel Plot of Articles (Artwork)

Figure S19 shows the Deeks' plot of articles classified under Document. The research done by A. R. Martin et al., has the smallest sample size as well as the lowest accuracy. The research done by Khan et al in 2018, has relatively large sample size while also having the highest accuracy value. The research conducted by Pereira et al. has the largest sample size and an accuracy value above the regression line.

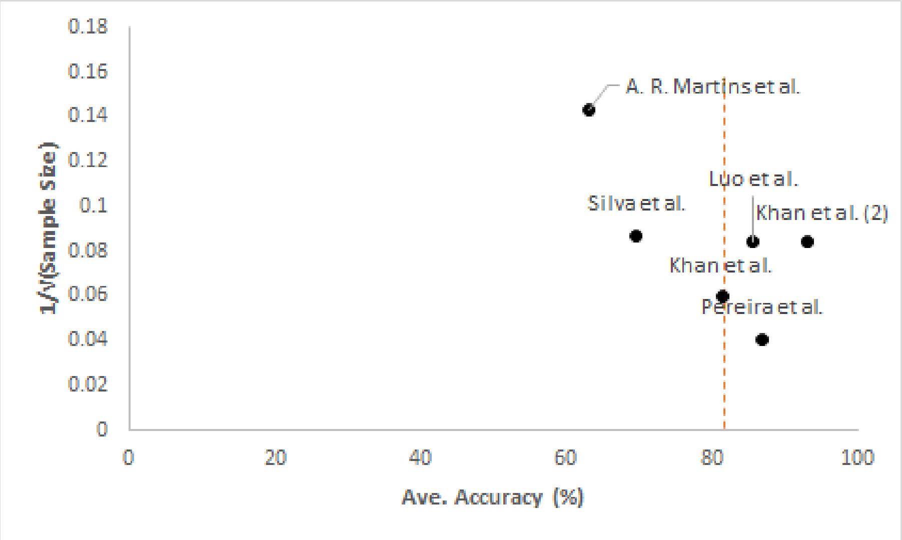

Figure S19. Deeks' Funnel Plot of Articles (Document)

Figure S20 shows the Deeks' plot of articles classified under Currency. The research done by Correia et al., has the highest accuracy but the smallest sample size. The research done by Baek et al, has the largest sample size, but the lowest accuracy. The research conducted by Kang et al. has relatively large sample size and an accuracy value well-above the regression line.

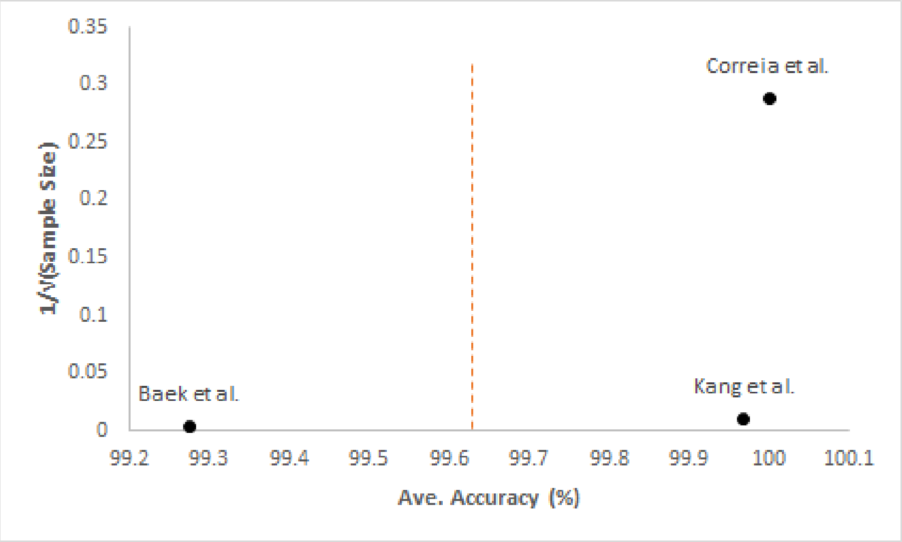

Figure S20. Deeks' Funnel Plot of Articles (Currency)

Figure S21 shows the Deeks' plot of topic classifications. The researches classified under currency has the largest sample size as well as the highest accuracy. The researches classified under photo has an accuracy above the regression line, however, has the smallest sample size. The researches classified under artwork has relatively large sample size but the lowest accuracy.

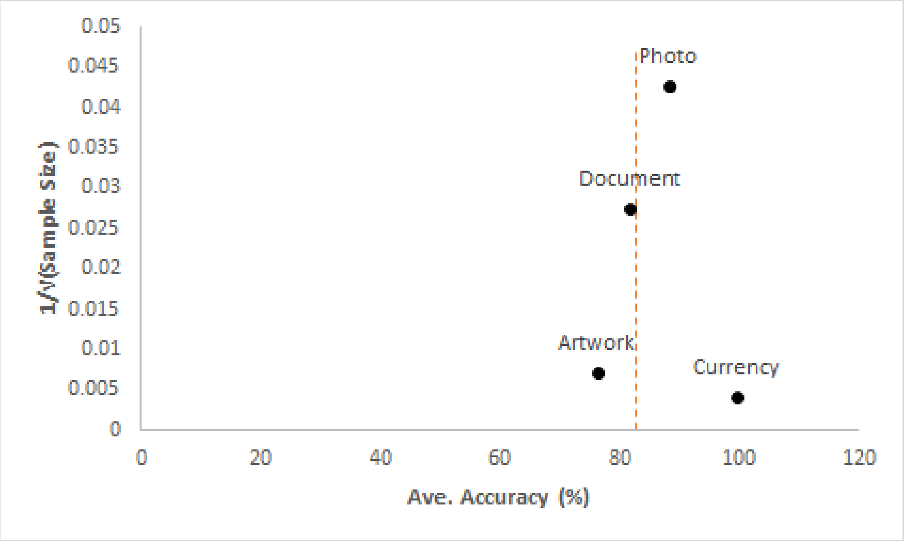

Figure S21. Deeks' Funnel Plot of Topic Classifications

Figure S22 shows the Deeks' plot of studies depending on the spectral range they selected in terms of its average accuracy. The studies that selected multiple ranges had the second to the largest sample size and had the highest accuracy value. VNIR, despite having the largest sample size, also had the lowest accuracy value.

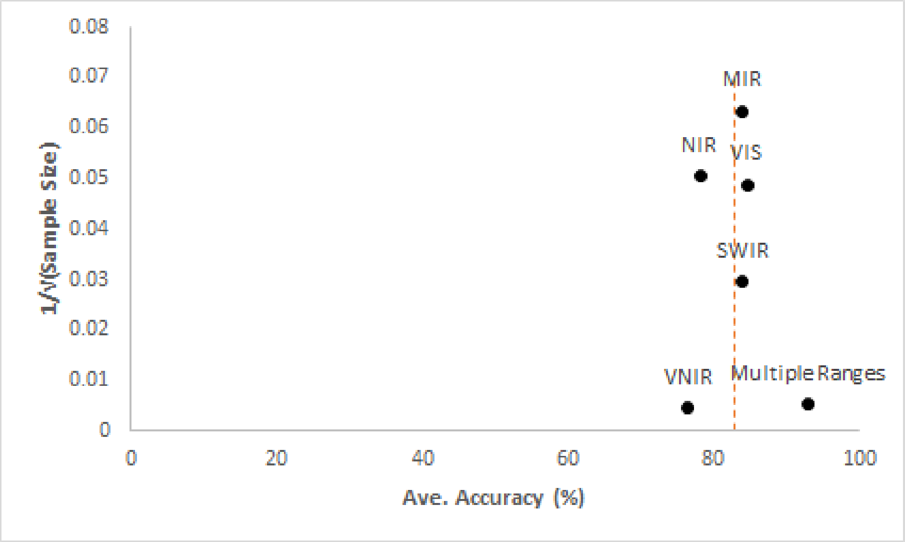

Figure S22. Deeks' Funnel Plot of Wavelengths

Figure S23 shows the Deeks' plot of different processing methods. The researches that used own algorithms and/or multiple algorithms in mix has the largest sample size and the highest accuracy value amongst all machine learning processes, followed by CNN with relatively large sample size and similar accuracy value and SVM with lowest accuracy and smallest sample size. In case of processing methods for dimension reduction, PCA and SSA both have relatively large sample size and low accuracy whereas PP has the highest accuracy but with relatively smaller sample size.

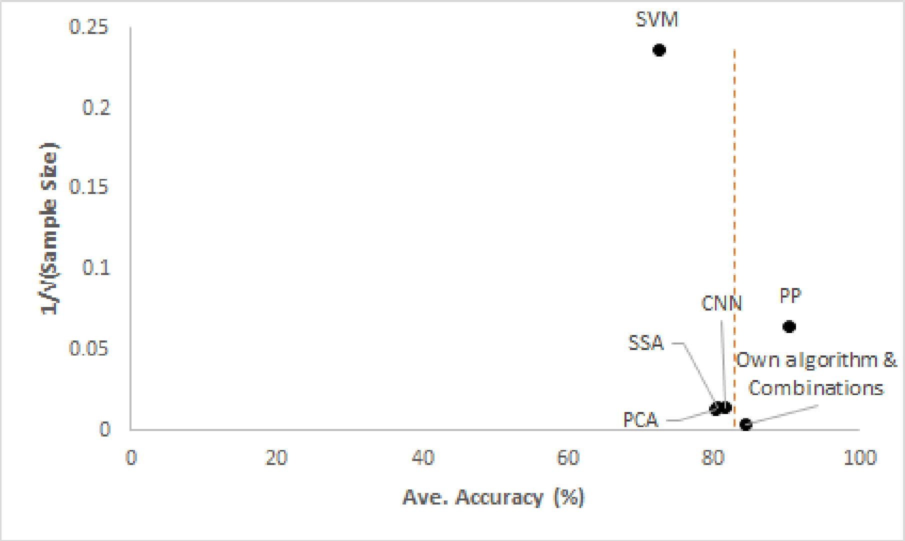

Figure S23. Deeks' Funnel of Processing Methods

Figure S24 shows the Deeks' plot of studies depending on the year they were published. The studies that were published in 2018 had the largest sample size and the highest accuracy value. Studies published in 2016, while not as high as those published in 2018, had relatively high accuracy values just above the regression line, along with a sample size similar to those published in 2018.

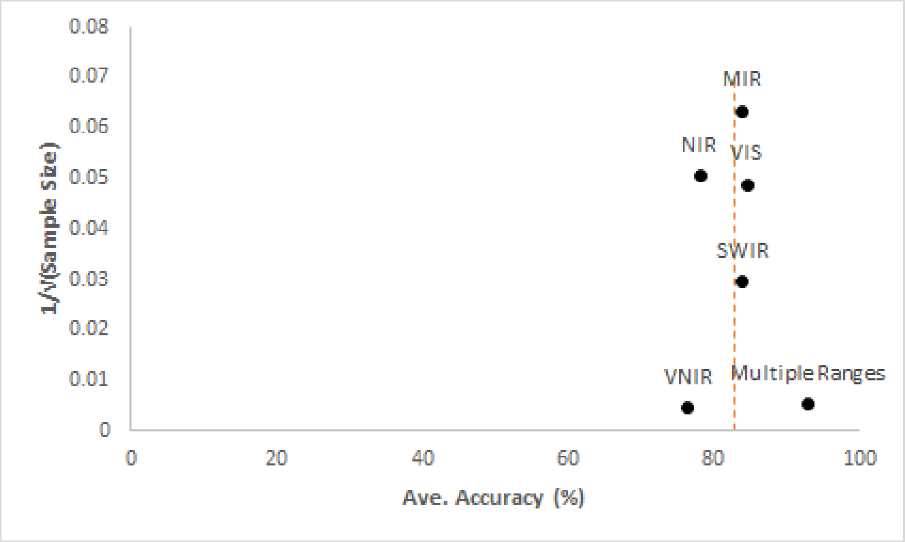

Figure S24. Deeks' Funnel Plot of Year Published

2. Supplementary Materials 2

Supplementary Materials 2 provides tables of calculations used in drafting of figures.

2.1 Forest Plots

Tables provided in this section includes calculations used in drafting forest plots.

Table S1. Forest Plot of Articles (Artwork)

Accuracy %	Polak et al.	Wang et al.	Grabowski et al.	Overall (Artwork)
Mean	72.5	74.025	80.7775	76.421
Std. Err.	5.5	5.784515969	6.535822283	3.502791411
Median	72.5	76.55	84.56	78.68
Mode	N/A	N/A	N/A	N/A
Std. Dev.	7.778174593	11.56903194	13.07164457	11.07679903
S. Var.	60.5	133.8425	170.8678917	122.6954767
Kurt	N/A	0.266847864	0.41646234	-0.984293139
Skew	N/A	-1.012378742	-1.176023587	-0.310120682
Range	11	26.2	28.33	32.76
Min	67	58.4	62.83	58.4
Max	78	84.6	91.16	91.16
Sum	145	296.1	323.11	764.21
Count	2	4	4	10
CL	10.78	11.3376513	12.81021167	6.865471165
Upper CI	83.28	85.3626513	93.58771167	83.28647116
Lower CI	61.72	62.6873487	67.96728833	69.55552884

Table S2. Forest Plot of Articles (Document)

Accuracy %	Silva et al.	Pereira et al.	Khan et al.	Khan et al. (2)	Luo et al.	A. R. Martins et al.	Overall (Document)
Mean	69.66666667	87	81.45	93.1	85.65	63	81.78235294
Std. Err.	13.86041526	3.45890156	3.046035675	5.1	3.35	0	3.236365464
Median	82	87.5	81.05	93.1	85.65	63	85
Mode	N/A	N/A	N/A	N/A	N/A	N/A	89
Std. Dev.	24.00694344	7.734339015	6.09207135	7.212489168	4.737615434	N/A	13.34387665
S. Var.	576.3333333	59.82	55.57666667	52.02	22.445	N/A	178.0590441
Kurt	N/A	0.271299606	2.91772859	N/A	N/A	N/A	4.331055894
Skew	-1.701671753	0.037256724	0.074145102	N/A	N/A	N/A	-1.792752584
Range	43	20.8	14.3	10.2	6.7	0	56.2
Min	42	76.7	74.7	88	82.3	63	42

Max	85	97.5	89	98.2	89	63	98.2
Sum	209	435	325.8	186.2	171.3	63	1390.3
Count	3	5	4	2	2	1	17
CL	27.1664139	6.779447057	5.970229923	9.996	6.566	0	6.34327631
Upper CI	96.83308057	93.77944706	87.42022992	103.096	92.216	63	88.12562925
Lower CI	42.50025276	80.22055294	75.47977008	83.104	79.084	63	75.43907663

Table S3. Forest Plot of Articles (Currency)

Accuracy %	Baek et al.	Kang et al.	Correia et al.	Overall (Currency)
Mean	99.275	99.96875	100	99.6296875
Std. Err.	0.615	0	0	0.324056473
Median	99.275	99.96875	100	99.929375
Mode	N/A	N/A	N/A	N/A
Std. Dev.	0.869741341	N/A	N/A	0.648112946
S. Var.	0.75645	N/A	N/A	0.420050391
Kurt	N/A	N/A	N/A	3.895029698
Skew	N/A	N/A	N/A	-1.969806623
Range	1.23	0	0	1.34
Min	98.66	99.96875	100	98.66
Max	99.89	99.96875	100	100
Sum	198.55	99.96875	100	398.51875
Count	2	1	1	4
CL	1.2054	0	0	0.635150687
Upper CI	100.4804	99.96875	100	100.2648382
Lower CI	98.0696	99.96875	100	98.99453681

Table S4. Forest Plot of Articles (Photo)

Accuracy %	Tournié et al.	Overall (Photo)
Mean	88.26666667	88.26666667
Std. Err.	4.141792419	4.141792419
Median	86	86
Mode	N/A	N/A
Std. Dev.	7.173794905	7.173794905
S. Var.	51.46333333	51.46333333

Kurt	N/A	N/A
Skew	1.279893812	1.279893812
Range	13.8	13.8
Min	82.5	82.5
Max	96.3	96.3
Sum	264.8	264.8
Count	3	3
CL	8.117913142	8.117913142
Upper CI	96.38457981	96.38457981
Lower CI	80.14875352	80.14875352

Table S5. Forest Plot of Topic Classifications

Accuracy %	Artwork	Document	Currency	Photo	Overall
Mean	76.421	81.78235294	99.6296875	88.26666667	82.87731618
Std. Err.	3.502791411	3.236365464	0.324056473	4.141792419	2.254786181
Median	78.68	85	99.929375	86	84.80
Mode	N/A	89	N/A	N/A	89
Std. Dev.	11.07679903	13.34387665	0.648112946	7.173794905	13.14754976
S. Var.	122.6954767	178.0590441	0.420050391	51.46333333	172.8580646
Kurt	-0.984293139	4.331055894	3.895029698	#DIV/0!	1.594722517
Skew	-0.310120682	-1.792752584	-1.969806623	1.279893812	-1.06696956
Range	32.76	56.2	1.34	13.8	58
Min	58.4	42	98.66	82.5	42.00
Max	91.16	98.2	100	96.3	100.00
Sum	764.21	1390.3	398.51875	264.8	2817.83

Count	10	17	4	3	34
CL	6.865471165	6.34327631	0.635150687	8.117913142	4.419380915
Upper CI	83.28647116	88.12562925	100.2648382	96.38457981	87.29669709
Lower CI	69.55552884	75.43907663	98.99453681	80.14875352	78.45793526

Table S6. Forest Plot of Wavelengths

**Accuracy %**	**MIR**	**SWIR**	**NIR**	**VNIR**	**VIS**	**Multiple Ranges**	**Overall**
Mean	84	83.98714286	78.14285714	76.49833333	84.6	92.86575	82.87731618
Std. Err.	8.976822006	4.116403142	6.680884499	6.205836187	3.668878121	2.694429079	2.254786181
Median	87.5	86	82.00	76.55	85.15	90.00	84.80
Mode	N/A	N/A	N/A	N/A	N/A	N/A	89
Std. Dev.	15.54831181	10.89097901	17.67595892	15.20113209	8.986879325	6.024926582	13.14754976
S. Var.	241.75	118.6134238	312.4395238	231.0744167	80.764	36.29974031	172.8580646
Kurt	N/A	2.149042886	3.863733555	-0.41971063	-0.72147973	-2.869114303	1.594722517
Skew	-0.961642223	-1.289029506	-1.53189416	0.422637134	0.36322826	0.451891206	-1.06696956
Range	30.5	33.47	58	41.49	23.5	13.26875	58
Min	67	62.83	42.00	58.40	74.70	86.70	42.00
Max	97.5	96.3	100.00	99.89	98.20	99.97	100.00
Sum	252	587.91	547.00	458.99	507.60	464.33	2817.83
Count	3	7	7	6	6	5	34
CL	17.59457113	8.068150159	13.09453362	12.16343893	7.191001117	5.281080995	4.419380915
Upper CI	101.5945711	92.05529302	91.23739076	88.66177226	91.79100112	98.146831	87.29669709
Lower CI	66.40542887	75.9189927	65.04832353	64.33489441	77.40899888	87.584669	78.45793526

Table S7. Forest Plot of Processing Methods

**Accuracy %**	**CNN**	**Own algorithm & Combinations**	**PCA**	**PP**	**SSA**	**SVM**	**Overall**
Mean	81.53333333	84.43835526	80.35714286	90.4	80.6	72.5	82.87731618
Std. Err.	11.93556776	3.403081432	2.662066611	7.1	0	5.5	2.254786181
Median	88	82	76.70	90.40	80.60	72.50	84.80
Mode	N/A	N/A	N/A	N/A	N/A	N/A	89
Std. Dev.	20.67300978	14.83368806	7.043166225	10.04091629	N/A	7.778174593	13.14754976
S. Var.	427.3733333	220.0383014	49.60619048	100.82	N/A	60.5	172.8580646
Kurt	N/A	2.698223424	-2.475768282	N/A	N/A	N/A	1.594722517
Skew	-1.269897956	-1.523059188	0.286101133	N/A	N/A	N/A	-1.06696956

Range	39.8	58	16.5	14.2	0	11	58
Min	58.4	42.00	72.50	83.30	80.60	67.00	42.00
Max	98.2	100.00	89.00	97.50	80.60	78.00	100.00
Sum	244.6	1604.33	562.50	180.80	80.60	145.00	2817.83
Count	3	19	7	2	1	2	34
CL	23.39371281	6.670039606	5.217650557	13.916	0	10.78	4.419380915
Upper CI	104.9270461	91.10839487	85.57479341	104.316	80.6	83.28	87.29669709
Lower CI	58.13962052	77.76831566	75.1394923	76.484	80.6	61.72	78.45793526

Table S8. Forest Plot of Year Published.

Accuracy %	2014	2015	2016	2017	2018	2019	Title 6
Mean	69.66666667	82.85	84.29759615	78.01833333	96.95	63	82.87731618
Std. Err.	13.86041526	2.662674094	3.095902639	4.706235875	2.26423497	0	2.254786181
Median	82.00	84.50	84.60	78.68	98.66	63.00	84.80
Mode	N/A	89	N/A	N/A	N/A	N/A	89
Std. Dev.	24.00694344	6.522192883	11.16243571	11.5278765	5.06298331	N/A	13.14754976
S. Var.	576.3333333	42.539	124.599971	132.8919367	25.6338	N/A	172.8580646
Kurt	N/A	-2.159876846	1.271116475	-1.620732148	4.533734972	N/A	1.594722517
Skew	-1.701671753	-0.46789382	-0.80758345	-0.186150134	-2.106646909	#DIV/0!	-1.06696956
Range	43	14.3	41.56875	28.33	12	0.00	58
Min	42.00	74.70	58.40	62.83	88.00	63.00	42.00
Max	85.00	89.00	99.97	91.16	100.00	63.00	100.00
Sum	209.00	497.10	1095.87	468.11	484.75	63.00	2817.83
Count	3	6	13	6	5	1	34
CL	27.1664139	5.218841225	6.067969172	9.224222315	4.437900541	0	4.419380915
Upper CI	96.83308057	88.06884123	90.36556533	87.24255565	101.3879005	63	87.29669709
Lower CI	42.50025276	77.63115877	78.22962698	68.79411102	92.51209946	63	78.45793526

2.2 Deeks' Funnel Plots

Tables provided in this section includes calculations used in drafting Deeks' funnel plots.

Table S9. Deeks' Funnel Plot of Articles (Artwork)

Regression Statistics	
Multiple R	0.531512128
R Square	0.282505143
Adjusted R Square	-0.434989715
Standard Error	0.147841085
Observations	3

Table S10. Deeks' Funnel Plot of Articles (Document)

Regression Statistics	
Multiple R	0.692842444
R Square	0.480030652
Adjusted R Square	0.350038315
Standard Error	0.027745949
Observations	6

Table S11. Deeks' Funnel Plot of Articles (Currency)

Regression Statistics	
Multiple R	0.549671975
R Square	0.30213928
Adjusted R Square	-0.39572144
Standard Error	0.191804587
Observations	3

Table S12. Deeks' Funnel Plot of Topic Classifications

Regression Statistics	
Multiple R	0.135900497
R Square	0.018468945
Adjusted R Square	-0.472296582
Standard Error	0.022040615
Observations	4

Table S13. Deeks' Funnel Plot of Wavelengths

Regression Statistics	
Multiple R	0.172025727
R Square	0.029592851
Adjusted R Square	-0.213008937
Standard Error	0.027271012
Observations	6

Table S14. Deeks' Funnel Plot of Processing Methods

Regression Statistics	
Multiple R	0.613184764
R Square	0.375995555
Adjusted R Square	0.219994444
Standard Error	0.079397525
Observations	6

Table S15. Deeks' Funnel Plot of Year Published

Regression Statistics

Multiple R	0.915953745
R Square	0.838971263
Adjusted R Square	0.798714079
Standard Error	0.023739548
Observations	6

2.3 Deeks' Funnel Plots p value

P value is used in evaluation of data heterogeneity. Tables provided in this section includes calculations used to acquire p value.

Table S16. P value of Articles (Artwork)

ANOVA

	df	*SS*	*MS*	*F*	*p value*
Regression	1	0.008605931	0.008605931	0.393738212	0.643247997
Residual	1	0.021856986	0.021856986		
Total	2	0.030462917			

Table S17. P value of Articles (Document)

ANOVA

	df	*SS*	*MS*	*F*	*p value*
Regression	1	0.002842827	0.002842827	3.69276115	0.127029139
Residual	4	0.003079351	0.000769838		
Total	5	0.005922177			

Table S18. P value of Articles (Currency)

ANOVA

	df	*SS*	*MS*	*F*	*p value*
Regression	1	0.015927823	0.015927823	0.43295069	0.629505422
Residual	1	0.036789	0.036789		
Total	2	0.052716823			

Table S19. P value of Topic Classifications

ANOVA

	df	*SS*	*MS*	*F*	*p value*
Regression	1	1.82817E-05	1.82817E-05	0.037632931	0.864099503
Residual	2	0.000971577	0.000485789		
Total	3	0.000989859			

Table S20. P value of Wavelengths

ANOVA

	df	*SS*	*MS*	*F*	*p value*
Regression	1	9.07184E-05	9.07184E-05	0.121981174	0.744506775
Residual	4	0.002974832	0.000743708		

Total	5	0.003065551			

Table S21. P value of Processing Methods

ANOVA

	df	*SS*	*MS*	*F*	*p value*
Regression	1	0.015193889	0.015193889	2.410210747	0.195500227
Residual	4	0.025215868	0.006303967		
Total	5	0.040409756			

Table S22. P value of Year Published

ANOVA

	df	*SS*	*MS*	*F*	*p value*
Regression	1	0.01174488	0.01174488	20.8402868	0.010298818
Residual	4	0.002254265	0.000563566		
Total	5	0.013999145			
